# Multimodal therapeutic strategies against gastric cancer: from conventional treatments to tumor microenvironment targeting

**DOI:** 10.3389/fimmu.2025.1623588

**Published:** 2025-09-04

**Authors:** Xi Yuan, Changxian Chen, Yixuan Pang, Xuanzhi Wang, Tingting Yang, Anying Long, Na Liang, Ying Yang, Chunming Li

**Affiliations:** ^1^ Department of Pathology, Zunyi Medical University, Zunyi, Guizhou, China; ^2^ Department of Pathology, Affiliated Hospital of Zunyi Medical University, Zunyi, Guizhou, China; ^3^ The First Clinical Institute, Zunyi Medical University, Zunyi, Guizhou, China

**Keywords:** gastric cancer, tumor immune microenvironment, heterogeneity of cell components, metabolic reprogramming, immunotherapy

## Abstract

Gastric cancer is one of the most common malignant tumors of the digestive system, with persistently high global morbidity and mortality rates. The multi-level heterogeneity of the gastric cancer tumor immune microenvironment (TIME) is closely associated with treatment efficacy and prognosis. This heterogeneity is reflected not only in the types and functions of various cells within the microenvironment but also in multiple aspects such as molecular profiles, metabolic pathways, and the spatial distribution of tumor cells. Currently, the interaction between gastric cancer and its microenvironment, as well as the resulting immune evasion, has become a research hotspot. This article reviews the role of cellular heterogeneity and metabolic reprogramming in the gastric cancer Tumor Immune Microenvironment (TIME) in reshaping the immune microenvironment, and summarizes traditional therapies alongside existing and potential microenvironment-modulating treatment strategies.

## 1 Introduction

Gastric cancer is a common malignant tumor of the digestive system, with morbidity and mortality rates ranking among the highest worldwide (1). Due to its high invasiveness, susceptibility to metastasis, and drug resistance, traditional treatment methods face significant challenges. Surgical resection combined with adjuvant chemotherapy or chemoradiotherapy remains the cornerstone of treatment. While surgery swiftly eradicates localized disease, it is inadequate against systemic micrometastases. Chemotherapy, with its systemic reach, is backed by robust evidence and extensive clinical experience; however, its efficacy is tempered by significant toxicity and tumor heterogeneity, limiting overall benefit. Radiotherapy can effectively reduce tumor burden, yet it often harms surrounding healthy tissues and carries a risk of treatment-related complications (2–6). Systemic antineoplastic therapy remains the cornerstone for advanced, unresectable, or metastatic gastric cancer (4). The effectiveness of these treatments is often constrained by a critical barrier—the immunosuppressive tumor immune microenvironment (TIME) (4, 7). The tumor immune microenvironment (TIME) is a dynamic and complex ecosystem consisting of various heterogeneities, including immune cells, the extracellular matrix, soluble factors (such as cytokines, chemokines, etc.), metabolites, and their spatial distribution. TIME can modulate tumor progression in a bidirectional manner through immunosuppression or immunoactivation, and is closely associated with patient prognosis (8, 9). The stomach’s prolonged exposure to dietary antigens and its abundant vascularization create a unique milieu. Under the influence of Helicobacter pylori infection, high salt intake, alcohol exposure, genetic predisposition, and other factors, chronic inflammation not only induces immunosuppression but also progressively sculpts and intensifies the complex TIME throughout gastric carcinogenesis (10, 11).

Given the constraints imposed by the TIME on conventional therapies, research has increasingly turned toward precise modulation of the tumor microenvironment, with targeted agents and immunotherapies emerging as major areas of interest. Targeted therapies exploit tumor-specific markers to selectively eliminate cancer cells while sparing normal tissue, thereby enhancing efficacy and minimizing adverse effects. In gastric cancer, agents such as the anti-HER2 antibody trastuzumab, the anti-angiogenic drugs ramucirumab and apatinib, and the CLDN18.2-directed monoclonal antibody zolbetuximab have ushered in an era of personalized treatment. However, their applicability is limited to biomarker-positive patients, and the development of resistance remains a significant hurdle (6, 12, 13). Immunosuppression is now recognized as a hallmark of malignancy, and immunotherapy has delivered notable advances in gastric cancer care. By reversing tumor-induced immune inhibition, these approaches reactivate endogenous anti-tumor immunity, often with manageable toxicity profiles. Key strategies include immune checkpoint inhibitors against PD-1/PD-L1 and CTLA-4, adoptive cell therapies, and therapeutic cancer vaccines. Yet, these modalities frequently require prolonged treatment courses and exhibit marked inter-patient variability in response (6, 13).

The shortcomings of current treatments arise from an incomplete understanding of the complex, gastric cancer–specific TIME. Therefore, a multi-level analysis of the heterogeneity of immune regulation within the gastric cancer TIME, along with an exploration of changes in immune cell characteristics, functional remodeling, metabolic factors, and signaling pathways, is crucial for uncovering the mechanisms underlying gastric cancer TIME remodeling.

With the development of cutting-edge technologies such as single-cell transcriptomics, spatial transcriptomics, and mass spectrometry imaging, our understanding of the cellular components, molecular regulation, and metabolic reprogramming within the gastric cancer TIME has become more refined. This review systematically examines contemporary advances in the heterogeneity of the gastric cancer TIME and associated intervention strategies. It explores the pivotal roles of diverse cellular subsets—such as M2-polarized TAMs, MDSCs, CAFs, and Tregs—and the impact of metabolic reprogramming on tumor progression. Additionally, it briefly summarizes conventional gastric cancer therapies and highlights recent developments in novel immunotherapeutic and combination regimens, with the aim of informing the future optimization of precision treatment approaches.

## 2 TIME heterogeneity in gastric cancer

### 2.1 Cellular composition heterogeneity

**Figure 1:**
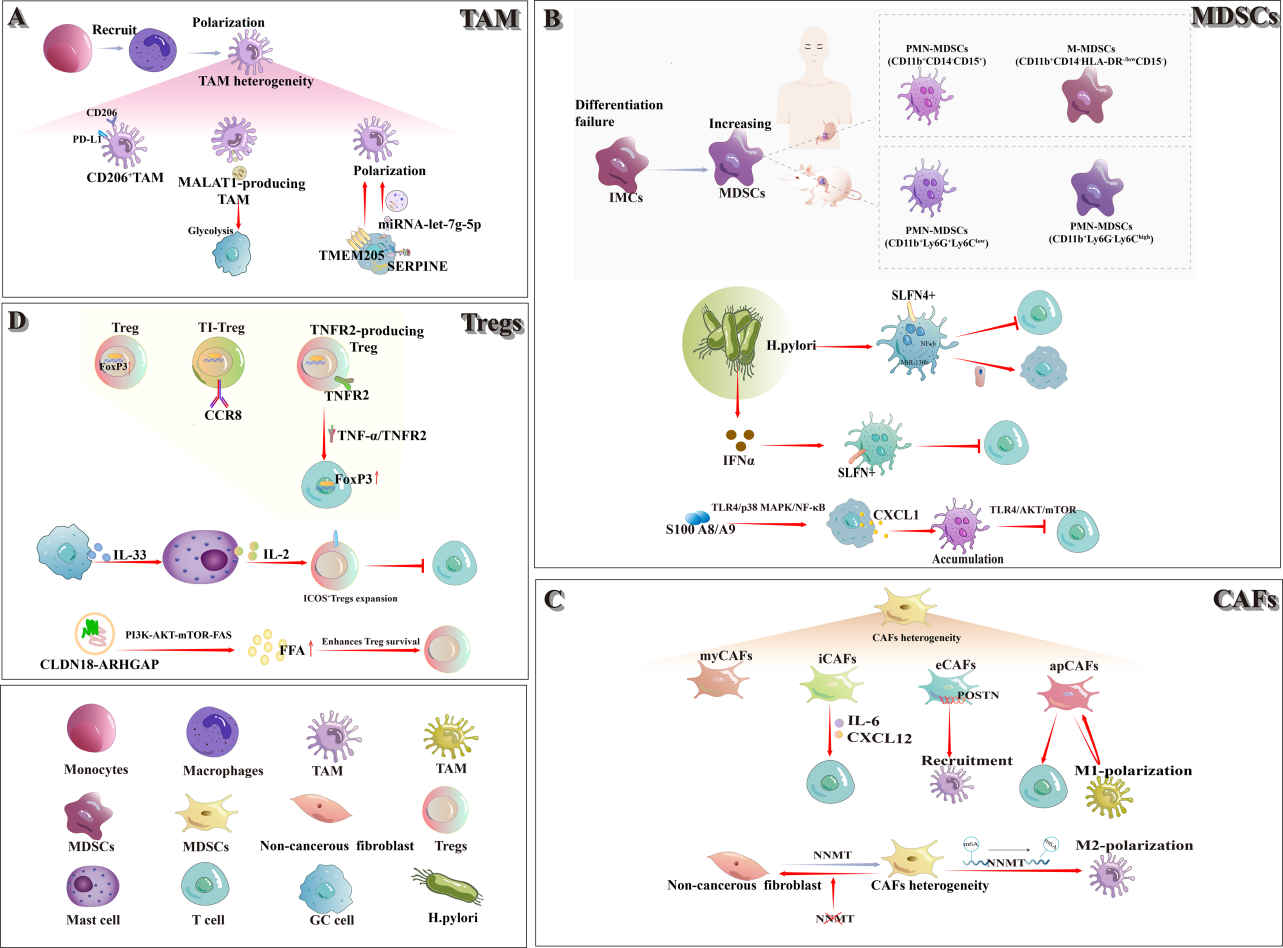
In the TIME of gastric cancer, the classical immunosuppressive cell populations comprise TAMs, MDSCs, CAFs, and Tregs. **(A)** TAMs: The majority of TAMs originate from the polarization of recruited monocytes, exhibit a heterogeneous population. CD206^+^ M2-TAMs notably express high levels of PD-L1. Exosomal MALAT1 released by M2-TAMs enhances glycolysis in gastric cancer cells. The transmembrane protein TMEM205 drives M2 polarization of TAMs, and tumor-derived SERPINE regulates exosomal let-7g-5p to further promote M2-TAM polarization. **(B)** MDSCs: Failure of IMCs to mature into functional myeloid lineages leads to an expansion of MDSCs. Two principal MDSC subsets—M-MDSCs and PMN-MDSCs—have been defined in both humans and mice. In chronic Helicobacter pylori infection, SLFN4^+^ MDSCs activate the NF-κB–miR-130b axis to inhibit T cells and drive epithelial carcinogenesis; elevated IFN-α further promotes SLFN4^+^ MDSC polarization and suppresses T-cell proliferation. Meanwhile, S100A8/A9 up-regulates CXCL1 in gastric cancer cells via the TLR4/p38 MAPK/NF-κB pathway, thereby enhancing PMN-MDSC recruitment and dampening CD8^+^T cell activity. **(C)** CAFs: CAFs are phenotypically diverse and functionally heterogeneous. Currently known subtypes include myCAFs, iCAFs, eCAFs, and apCAFs, among others. iCAFs secrete IL-6 and CXCL12 to modulate T-cell interactions; eCAFs—POSTN expression—promote M2-TAM recruitment; and apCAFs both enhance T-cell function and drive macrophage polarization, which in turn sustains apCAF formation. **(D)** Tregs: As the master transcription factor of Tregs, FoxP3 is markedly up-regulated within the tumor immune microenvironment. CCR8^+^ TI-Tregs display highly specific expression, making them an ideal therapeutic target. TNFR2^+^ Tregs regulate FoxP3 expression via the TNF-α/TNFR2 signaling axis. Gastric cancer–derived IL-33 drives mast cell secretion of IL-2, thereby expanding ICOS^+^Tregs and suppressing CD8^+^T-cell activity. Finally, the CLDN18-ARHGAP enhances FFA production and promotes Treg survival through activation of the PI3K-AKT-mTOR-FAS pathway.

In TIME, the heterogeneity of cellular components decisively influences tumor progression, and classic immunosuppressive populations predominate in gastric cancer (**Figure 1**). M2-TAMs, mainly derived from recruited monocytes, promote tumor cell proliferation, metastasis and drug resistance by secreting exosomes, proteins and chemokines. Meanwhile, MDSCs accumulate in large numbers due to differentiation blockade during tumorigenesis, further inhibiting T-cell function and constituting a key mechanism of tumor immune escape. Moreover, CAFs and Foxp3^+^ Tregs cooperatively establish an immunosuppressive network via multiple molecular pathways and cellular interactions. In recent years, application of cutting-edge technologies such as single-cell sequencing and spatial transcriptome analysis has further unveiled the dynamic changes and molecular characteristics of cell subsets in gastric cancer TIME, providing new theoretical foundations and potential targets for precise modulation of TIME and improved therapeutic outcomes.

#### 2.1.1 Classical immunosuppressive cell populations

##### 2.1.1.1 M2 tumor-associated macrophages

Macrophages, a subset of long-lived phagocytes integral to the innate immune system, are widely distributed across most tissues and serve as the first line of defense against pathogens. However, under the specific pathological context of TIME, macrophages undergo phenotypic transformation, lose their protective functions, and become TAMs (14). Within the TIME of gastric cancer, the majority of TAMs arise from the differentiation and polarization of monocytes recruited to the tumor site, with M2-TAMs representing the predominant subset within this compartment (15).

M2-TAMs exert their protumorigenic effects through the secretion of extracellular vesicles (EVs), bioactive proteins, and multiple chemokines, coupled with metabolic reprogramming mechanisms that collectively drive tumor cell proliferation, metastatic progression, and therapeutic resistance. Additionally, these macrophages facilitate gastric cancer pathogenesis through dual-phase modulation of the hepatic microenvironment: priming the premetastatic niche formation during early dissemination stages and subsequently fostering angiogenesis establishment in metastatic lesions (16). A recent investigation utilizing multiplex immunohistochemistry co-localized CD163 and CD206 markers on TAMs in gastric cancer specimens. Notably, the CD206^+^ M2-TAM subset was identified as a phenotypically distinct subpopulation exhibiting PD-L1 positivity (17). Wang et al. demonstrated that exosomes derived from M2-TAMs carry the long non-coding RNA MALAT1 and transfer it to gastric cancer cells. MALAT1 stabilizes δ-catenin and upregulates HIF-1α expression, thereby enhancing glycolysis and promoting the proliferation, metastasis, and chemoresistance of gastric cancer cells (18). Fu et al. demonstrated that transmembrane protein 205 (TMEM205) promotes proliferation and stemness, and enhances epithelial–mesenchymal transition (EMT), migration, and angiogenesis in gastric cancer cells. Moreover, TMEM205-induced polarization of M2-TAMs further accelerates gastric cancer progression (19). Recent studies have shown that SERPINE, a serine protease inhibitor derived from gastric cancer cells, serves as a major driver of gastric cancer growth and promoting M2-TAM polarization. Through autocrine activation of the JAK2/STAT3 signaling pathway to mediate the transfer of exosomal let-7g-5p, thereby facilitating this polarization (20).

##### 2.1.1.2 Myeloid-derived suppressor cells

MDSCs constitute a heterogeneous population predominantly comprising myeloid progenitor cells and immature myeloid cells (IMCs). Under physiological conditions, they are generated in the bone marrow and serve as precursors to macrophages, dendritic cells, and granulocytes. However, during tumorigenesis, IMCs frequently fail to complete their differentiation, resulting in an accumulation of MDSCs (21). In tumor-bearing mice, two principal MDSC subpopulations have been characterized: polymorphonuclear MDSCs (PMN-MDSCs) and monocytic MDSCs (M-MDSCs) (22). Among them, PMN-MDSCs are defined as CD11b^+^ Ly6G^+^ Ly6C^low^ cells, whereas M-MDSCs are defined as CD11b^+^ Ly6G^-^ Ly6C^high^ cells. In mice, CD49d can be used in place of Gr-1 to identify highly immunosuppressive MDSCs. Because humans lack Gr-1, the equivalent human subsets are defined as CD11b^+^ CD14^-^ CD15^+^ cells (or CD11b^+^ CD14^-^ CD66b^+^ cells) and CD11b^+^ CD14^-^ HLA-DR^-/low^ CD15^-^ cells, respectively (23).

Within gastric cancer, MDSC subpopulations exhibit considerable complexity. Ding et al. (24) demonstrated that, during Helicobacter pylori infection, bone marrow–derived Schlafen4^+^ (SLFN4^+^) MDSCs migrate to the stomach and activate the NF-κB pathway, leading to robust induction of MiR-130b. MiR-130b not only sustains persistent NF-κB activation but also contributes to TIME formation by suppressing T-cell responses and directly stimulating epithelial cell proliferation, thereby promoting metaplasia and cancer progression. Subsequent studies have demonstrated that Toll-like receptor 9 (TLR9) upregulates IFNα expression in gastric epithelial cells and plasmacytoid dendritic cells (pDCs) during Helicobacter pylori infection, thereby facilitating the polarization of SLFN^+^ MDSCs and suppressing T cell proliferation (25). S100 A8/A9 heterodimer upregulates CXCL1 expression in gastric cancer cells via the TLR4/p38 MAPK/NF-κB pathway, thereby driving PMN-MDSC accumulation in the tumor microenvironment. Furthermore, PMN-MDSCs exploit S100 A8/A9 to inhibit CD8^+^ T-cell glycolysis, proliferation and TNF-α/IFN-γ production through the TLR4/AKT/mTOR pathway, ultimately inducing T-cell exhaustion (26).

##### 2.1.1.3 Cancer-associated fibroblasts

Under normal physiological conditions, fibroblasts are classified as mesenchymal cells. However, their phenotypic diversity, functional heterogeneity, and lack of specific markers make it difficult to precisely determine their origin and function. During cancer development, CAFs are defined as non-epithelial, non-cancerous, non-endothelial, non-immune fibroblasts located within or adjacent to the tumor, which may derive from tissue-resident fibroblasts or from pancreatic and hepatic stellate cells (27). Years of investigation have identified two principal CAF subtypes with shared genetic profiles: myofibroblastic CAFs (myCAFs) and inflammatory CAFs (iCAFs). Paracrine factors secreted by epithelial cells—including TGF-β, IL-1α, and PDGF—play a central role in CAF reprogramming. Notably, the TGF-β and IL-1α axes have been shown to preferentially drive the differentiation of myCAFs and iCAFs, respectively (28).

Li et al. (29) identified four CAF subpopulations with distinct properties in gastric cancer. Among these, iCAFs and extracellular matrix CAFs (eCAFs) engage in bidirectional signaling with neighboring immune cell subpopulations in the tumor microenvironment. Specifically, iCAFs modulate T-cell behavior by secreting IL-6 and CXCL12, whereas eCAFs promote M2-TAM recruitment through elevated periostin (POSTN) expression. Another study demonstrated that the fat content and obesity-related gene (FTO) promote M2-TAM polarization by regulating m6A-dependent demethylation of Nicotinamide N-methyltransferase (NNMT) in CAFs, thereby driving gastric cancer progression (30). In addition, Eckert et al. (31) identified NNMT as a key metabolic regulator of CAF differentiation and showed that inhibition of its activity can reverse the CAF phenotype. A recent study identified antigen-presenting CAFs (apCAFs) in gastric cancer that not only enhance T cell activation, cytotoxicity, and proliferation—thereby augmenting T cell–mediated antitumor immunity—but also drive macrophage polarization toward a proinflammatory phenotype. These polarized macrophages, in turn, reinforce the formation of apCAFs, establishing a positive feedback loop that further amplifies antitumor immune responses (32).

##### 2.1.1.4 Forkhead box P3^+^ regulatory Treg cells

Regulatory T cells (Tregs) are an immunosuppressive subset of CD4^+^ T cells that are essential for maintaining immune homeostasis. FoxP3, the master transcription factor governing Treg differentiation, development and functional integrity, is critical to their suppressive activity. Within the TIME, Tregs are typically highly activated and exhibit potent immunosuppressive functions, characterized by upregulated expression levels of FoxP3 and Helios (33, 34). A recent study reported that hemokine (C-C motif) receptor 8 (CCR8) is highly expressed on effector tumor-infiltrating regulatory T cells (TI-Tregs), but its expression remains relatively low on peripheral Tregs and conventional T cells in both mice and humans, rendering CCR8 an ideal candidate for the selective targeting of TI-Tregs (35).

In gastric cancer, Tregs exert immunosuppressive effects via multiple mechanisms that promote tumor progression. Studies have demonstrated that TNFR2^+^ Tregs accumulate in the tumor microenvironment as the disease advances, and their level of infiltration can serve as a prognostic marker. Moreover, *in vitro* experiments reveal that TNF-α/TNFR2 signaling upregulates Foxp3 expression in CD4^+^CD25^+^ T cells and increases TGF-β secretion by Tregs, further enhancing their immunosuppressive capacity (36). Lv et al. (37) demonstrated that gastric cancer–derived IL-33 induces activation of the p38 MAPK pathway in mast cells, leading to IL-2 secretion and consequent expansion of ICOS^+^ Tregs. This expansion enhances the immunosuppressive capacity of Tregs while attenuating anti-tumor CD8^+^ T-cell activity, thereby driving gastric cancer progression. Wang et al. (38) identified the CLDN18-ARHGAP fusion gene as a primary source of immunogenic neoepitopes. As a pivotal regulator of the tumor immune microenvironment, this fusion enhances Treg survival by activating the PI3K-AKT-mTOR-FAS signaling cascade and augmenting free fatty acid (FFA) production, thereby promoting the establishment of the gastric cancer TIME.

#### 2.1.2 Novel research and discoveries

##### 2.1.2.1 Single cell sequencing reveals subpopulations dynamics

Since Single-Cell RNA Sequencing (scRNA-seq) was designated Technology of the Year by Nature Methods in 2013 (39), scRNA-seq has been widely used to study TIME heterogeneity in gastric cancer. Sathe et al. (40) performed single-cell transcriptome sequencing in patients with gastric cancer and intestinal metaplasia, revealing significant enrichment of stromal cells, macrophages, dendritic cells (DC) and T cells in the gastric cancer TIME, accompanied by an extensive cell-reprogramming phenomenon. By sequencing the single-cell transcriptomes of gastric cancer and adjacent mucosa (AM) samples, the researchers revealed pronounced intra- and intertumoral heterogeneity among tumor epithelial cells, whereas CAFs exhibited predominantly intratumoral heterogeneity. In addition, four CAF subgroups with distinct characteristics were identified; although they resemble resident fibroblasts in the AM, they display enhanced pro-tumor activity (29). Bian et al. (41) utilized the optimized single-cell multi-genomic sequencing method (scTrio-seq3) to map the DNA methylation landscape of gastric cancer at single-cell resolution and to identify candidate DNA methylation biomarkers. Moreover, they systematically delineated the relationships among genetic lineage, DNA methylation patterns, and transcriptional clusters at the single-cell level, providing a more detailed analysis of the molecular features underlying intratumoral heterogeneity and differentiation status in human gastric cancer than traditional approaches. Some researchers performed scRNA-seq and single-cell TCR sequencing (scTCR-seq) on patients with newly treated gastric adenocarcinoma across various Lauren subtypes. Their findings showed that intratumoral heterogeneity (ITH) serves as a prognostic marker for recurrence. Compared with ITH-L tumors, ITH-H tumors exhibited pronounced immunosuppressive features, including a reduced number of activated CD8+ T cells, an increased number of depleted CD8+ T cells, and marked polarization of M2-TAMs (42). Through scRNA-seq analysis of malignant cells in gastric cancer ascites, we identified a gastric-dominant subtype—predominantly composed of gastric cell lines—and a GI-mixed subtype—characterized by a combination of gastric- and colon-like cells. When integrated with immune-infiltration data from public databases, the superior prognosis of GI-mixed tumors appears linked to a more effective antitumor immune response, hallmarked by high levels of polarized B cells and M1-TAMs, low levels of polarized fibroblasts and M2-TAMs, and elevated cytolytic activity (43).

##### 2.1.2.2 Spatial heterogeneity

Spatial transcriptomics (ST) sequencing technology was designated Technology of the Year in 2020. Its principal advantage lies in its ability to profile gene expression while preserving spatial context within tissues, offering significant potential to elucidate complex, heterogeneous microenvironments (44, 45). Several recent pioneering studies have further broadened the applications of this technology. For instance, Joakim Lundeberg’s group (46) introduced a depth‐generation model based on spatial expression data that fuses low-resolution ISC datasets with high-resolution histological images to infer super-resolution expression maps, enabling analysis of gene expression within fine-grained anatomical structures. Likewise, the Mingyao Li team (47) developed iStar, a hierarchical image-feature extraction method that integrates ST data with high-resolution histological images to predict spatial gene expression at super-resolution.

ST technology plays a critical role in gastric cancer research. Joseph J. Zhao’s team utilized it to perform spatial transcriptomic analysis on 67 samples. Their findings revealed significant differences in the molecular characteristics and immune composition between peritoneal metastasis and other types of metastasis (such as liver metastasis), suggesting that peritoneal metastasis may involve a distinct biological mechanism. These results further underscore the pivotal role of TIME in transluminal metastasis (48). In addition, Patrick Tan’s research team integrated spatial transcriptomic and single-cell sequencing data, revealing that gastric cancer exhibits intra-tumor heterogeneity (ITH) based on RNA expression, characterized by two distinct evolutionary trajectories. These trajectories are closely associated with molecular subtypes, clinical prognosis, and the stromal microenvironment, where the tumor-stroma interface demonstrates a unique ecological state mediated by TGF-β signaling. Furthermore, the study identified SOX9 as a potential intrinsically dispersed evolutionary driver (48). These findings not only deepen the understanding of gastric cancer heterogeneity but also offer new perspectives for the development of precise therapeutic strategies and spatial biomarkers.

### 2.2 Molecular and metabolic heterogeneity

**Figure 2:**
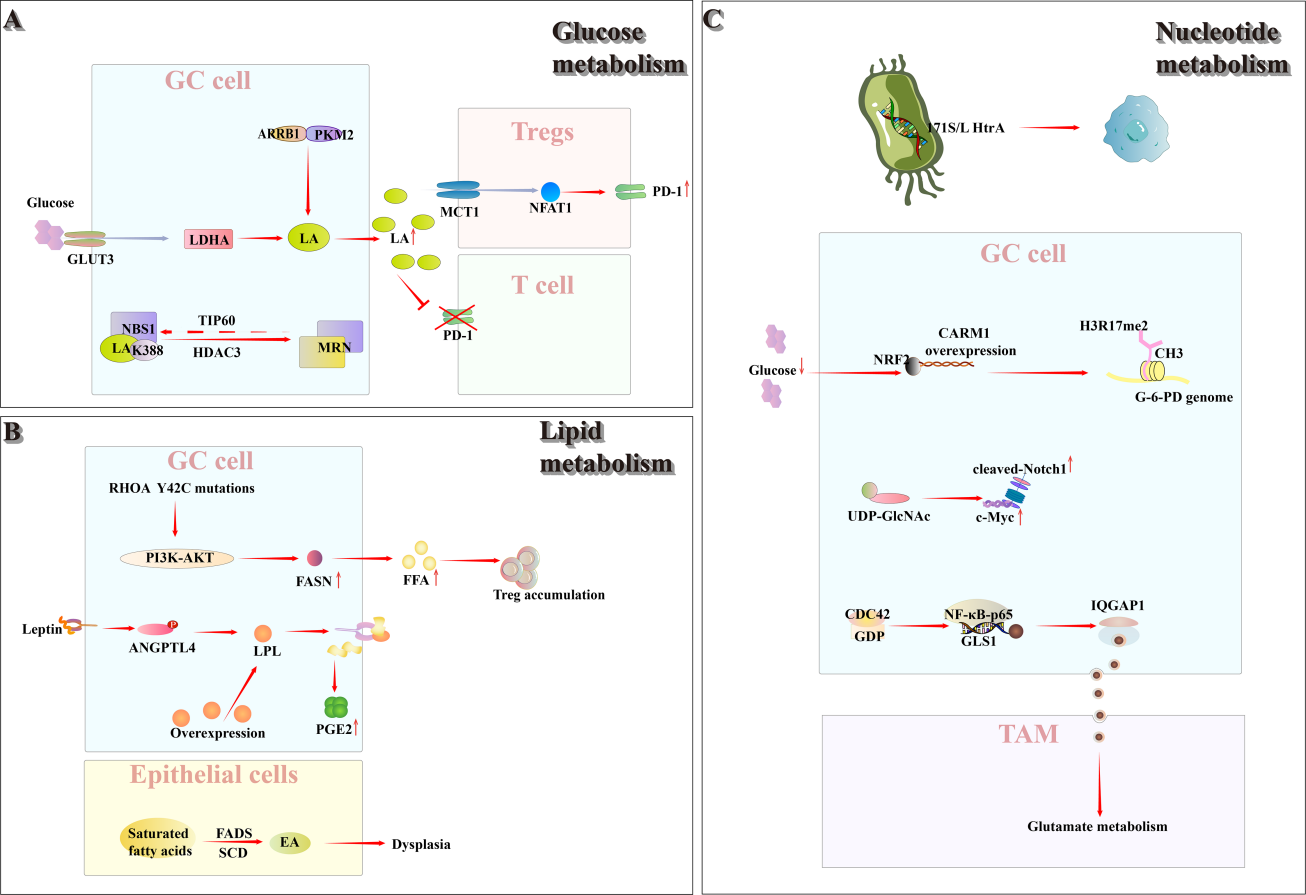
Within TIME, glucose, lipid, and nucleotide metabolism are each reprogrammed to varying degrees. **(A)** Glucose metabolism: Gastric cancer cells, via ARRB1, inhibit PKM2 tetramerization and up-regulate GLUT3, thereby reprogramming glucose metabolism toward aerobic glycolysis and increasing lactate production and secretion. Accumulated lactate drives dynamic lactylation of NBS1 at K388 (regulated by TIP60 and HDAC3) in cancer cells, promoting MRN complex assembly and altering DNA repair. Conversely, in the resulting low-glucose, high-lactate milieu, Tregs import lactate through MCT1, which activates NFAT1 signaling to up-regulate PD-1 and bolster their immunosuppressive function, while simultaneously suppressing PD-1 expression in effector T cells. **(B)** Lipid metabolism: In RHOA Y42C-mutant gastric cancer, FASN is overexpressed and PI3K–AKT signaling drives excessive FFA production, fostering Treg accumulation within the tumor microenvironment. Leptin-stimulated ANGPTL4 phosphorylation enhances lipoprotein lipase–mediated lipid uptake and thereby augments PGE_2_synthesis. Moreover, stearoyl-CoA desaturase–dependent desaturation generates EA, which promotes gastric epithelial dysplasia. **(C)** Nucleotide metabolism: The Helicobacter pylori 171S/L HtrA mutation—a cancer‐associated SNP unique to this bacterium—is strongly linked to gastric cancer progression. CARM1 is frequently overexpressed in gastric tumors; under low–glucose conditions outside cancer cells, activated NRF2 binds the CARM1 promoter, driving its transcription and markedly increasing H3R17me2 levels across the glucose-6-phosphate dehydrogenase locus. Concurrently, enhanced pyrimidine biosynthesis amplifies Notch signaling and transcriptionally upregulates c-Myc. Additionally, gastric cancer cells overexpress CDC42, which, via NF-κB p65 activation, promotes GLS1–containing microvesicle release, thereby modulating macrophage glutamine metabolism and skewing polarization toward an M2-TAM phenotype.

Molecular and metabolic heterogeneity also plays a critical role in the TIME, directly influencing tumor growth, metastasis, and drug resistance (**Figure 2**). Glucose metabolic reprogramming represents a major mechanism underlying TIME remodeling in gastric cancer. Under the Warburg effect, cancer cells predominantly rely on glycolysis for energy production even in the presence of sufficient oxygen, with markedly increased glucose uptake and utilization, leading to the accumulation of large amounts of lactic acid. Concurrently, lipid metabolic reprogramming is also crucial in gastric cancer, further promoting immune suppression within the TIME. Moreover, aberrant nucleotide metabolism weakens tumor immune surveillance by modulating multiple mechanisms, including MHC-I expression and antigen presentation. Emerging evidence indicates that exosomes function as intercellular signaling carriers capable of reprogramming both cancer cells and stromal cells within the microenvironment. Utilizing spatial metabolomic techniques, such as mass spectrometry imaging, significant metabolic differences between tumor and stromal regions have been identified, offering a novel perspective for the precise analysis of metabolic regulation within the TIME.

#### 2.2.1 Classical metabolic pathways

##### 2.2.1.1 Reprogramming of glucose metabolism in gastric cancer reshapes the TIME

Warburg’s seminal report, published 100 years ago, demonstrated that cancer tissue sections convert glucose to lactic acid despite sufficient oxygen availability; this phenomenon is now called the Warburg effect (49). Although glycolysis yields far less energy than oxidative phosphorylation, cancer cells nevertheless depend on it by consuming large quantities of glucose. To meet the metabolic demands of rapid proliferation, cancer cells frequently alter receptor-mediated signaling pathways through specific genetic mutations, thereby activating and upregulating nutrient-uptake mechanisms. As a result, their capacity for glucose uptake and utilization is markedly enhanced (50). In gastric cancer cells, cytoplasmic β-Arrestin1 (ARRB1) binds to pyruvate kinase M2 (PKM2), inhibiting PKM2 tetramerization, decreasing its enzymatic activity, and modulating metabolic flux, thereby shifting cellular metabolism from oxidative phosphorylation to aerobic glycolysis (51). In addition, studies have demonstrated that Glucose Transporter 3(GLUT3) expression is markedly up-regulated in gastric cancer cells and is inversely correlated with patient prognosis. GLUT3 may facilitate tumor growth and metastasis by modulating Lactate dehydrogenase A(LDHA) activity (52). Driven by the Warburg effect, lactic acid—the end product of glycolysis—accumulates at high levels within gastric cancer cells and is exported extracellularly, where it plays a pivotal role in promoting immune escape and reshaping the gastric cancer TIME.

Lactatic-driven lactoylation of NBS1K388 promotes the formation of the MRE11-RAD50-NBS1(MRN) complex, thereby enhancing DNA-repair capacity and mediating chemotherapy resistance. This modification is regulated by TIP60-mediated lactoylation and HDAC3-catalyzed delactylation, and elevated NBS1 lactoylation correlates with poor prognosis in patients undergoing neoadjuvant chemotherapy (53). In tumors characterized by high glycolytic flux—including gastric cancer—excessive glucose uptake by tumor cells generates a microenvironment of low glucose and high lactate. Regulatory T cells (Tregs) actively import lactate via monocarboxylate transporter 1 (MCT1), inducing nuclear translocation of activated nuclear factor of activated T cells 1 (NFAT1) and consequent upregulation of PD-1; while PD-1 expression in effector T cells is inhibited, that is, the PD-1 expression and its inhibitory activity of Tregs are enhanced, which leads to poor PD-1 blocking therapy (54).

##### 2.2.1.2 Reprogramming of lipid metabolism in gastric cancer reshapes the TIME

Lipid metabolic reprogramming is increasingly recognized as a hallmark of tumor cells (55). It plays an important role in the progression of gastric cancer. Kumagai et al. (56) found that gastric cancers harboring the RHOA Y42C mutation exhibited significant enrichment of fatty acid metabolism–related gene sets and upregulation of fatty acid synthase (FASN) expression. Further studies revealed that this mutation not only impaired chemokine recruitment but also enhanced free fatty acid (FFA) production in effector T cells via the PI3K–AKT signaling pathway. The resulting elevated FFA levels promoted Treg cell accumulation, thereby facilitating the formation of TIME; elevated FFA levels, in turn, promote regulatory T-cell (Treg) accumulation and thereby facilitate tumor immune microenvironment (TIME) formation. Furthermore, studies have demonstrated that elevated intracellular lipid levels are a hallmark of lymph node–positive (N+) gastric cancer. In N+ gastric cancer, leptin-induced phosphorylation of angiopoietin-like protein 4 (ANGPTL4) enhances lipid uptake via overexpressed lipoprotein lipase (LPL), thereby stimulating prostaglandin E2 (PGE2) production and ultimately facilitating lymph node metastasis (57). Oleic acid (EA), produced via stearoyl-CoA desaturase (SCD)-dependent fatty acid desaturation, promotes the proliferation and survival of dysplastic gastric epithelial cells, thereby establishing an energy-supply chain through carcinogenic fatty acid metabolism during gastric cancer development. This metabolic reprogramming contributes to the reorganization of the gastric cancer tumor immune microenvironment (TIME) (58). Recent studies have demonstrated that in gastric cancer patients the intestinal microbiota is disrupted, leading to aberrant lipid metabolism that facilitates tumor progression. A significant reduction in the abundance of short chain fatty acid (SCFA)–producing bacteria results in decreased butyrate levels. SCFAs bind specifically to Hydroxycarboxylic acid receptor 2(HCAR2, also called GPR109A), which selectively recognizes butyrate, inhibiting gastric cancer progression by enhancing CD8^+^ T-cell–mediated cytotoxicity, including that of CAR-Claudin 18.2^+^ CD8^+^ T cells (59).

##### 2.2.1.3 Reprogramming of nucleotide metabolism in gastric cancer reshapes the TIME

In pan-cancer, the hyperphysiological abundance and dysregulated metabolism of nucleotides in cancer cells not only support rapid proliferation and DNA repair, but also promote immune evasion and drug resistance through multiple mechanisms. For instance, cancer cells modulate MHC-I expression, antigen presentation, and immune-related signaling pathways (such as purine metabolism-mediated immune regulation), thereby attenuating the anti-cancer activity of various cells within the tumor TIME (60).

Multiple studies have demonstrated the critical role of nucleotide metabolism in gastric cancer development. Through comprehensive genomic analysis of Helicobacter pylori, Tang et al. (61) discovered the 171S/L HtrA mutation as a unique bacterial cancer-associated SNP demonstrating significant association with gastric cancer development and progression. In addition, the study found that coactivator associated arginine methyltransferase 1 Gene(CARM1) is overexpressed in gastric cancer. Under the low-glucose conditions in the tumor microenvironment, Nuclear factor erythroid 2-related factor 2 (NRF2) becomes activated and binds to the CARM1 promoter, thereby upregulating its expression, and then triggering CARM1-mediated hypermethylation of histone H3 methylated at R arginine 17 (H3R17me2) in the glucose-6-phosphate dehydrogenase gene body. This epigenetic modification redirects glucose carbon flux toward the pentose phosphate pathway, facilitating nucleotide synthesis (e.g., production of nucleotide precursors such as ribose-5-phosphate) and maintaining redox homeostasis, ultimately promoting the survival and growth of gastric cancer cells (62). Furthermore, pyrimidine biosynthesis not only potentiates Notch signaling but also upregulates c-Myc transcriptional activity, consequently elevating key glycolytic enzymes. Importantly, the enhanced expression of critical pyrimidine synthesis enzymes CAD and DHODH confers chemotherapy resistance in gastric malignancies through glycolytic pathway activation (63). In HER2-positive gastric cancer, tumor cells activate CDC42 signaling to induce phosphorylation of NF-κB p65, thereby promoting the secretion of GLS1-enriched microvesicles. This molecular mechanism coordinates macrophage glutamine metabolic remodeling, facilitates M2 polarization of tumor-associated macrophages, and enhances pro-angiogenic signaling pathways, collectively contributing to the development of trastuzumab resistance (64).

#### 2.2.2 Emerging mechanism: exosomes

Exosomes can deliver biologically active molecules to recipient cells, reprogramming the metabolism of both cancer cells and their surrounding stromal cells, and thereby promoting cancer progression, angiogenesis, metastasis, drug resistance, and immunosuppression (65).

Studies have shown that Ubiquitin‐Specific Protease 7 (USP7) promotes CAFs in the gastric cancer TIME to secrete miR-522–laden exosomes by regulating the deubiquitination of heterogeneous nuclear ribonucleoprotein A1 (hnRNPA1). miR-522 then targets Arachidonic Acid 15-Lipoxygenase (ALOX15), prevents lipid ROS accumulation, and thereby inhibits iron death in cancer cells (66). Multiple studies have demonstrated that exosomes induce M2-TAM polarization, thereby promoting gastric cancer progression. Exosomal circATP8A1 drives M2-TAM polarization by modulating the miR-1-3p/STAT6 axis (67). Likewise, exosomal let-7g-5p induces M2-TAM polarization via autocrine activation of the JAK2/STAT3 signaling pathway (20). In addition, Li et al. (68) demonstrated that exosomal Thrombospondin-1 (THBS1) derived from gastric cancer cells is downregulated in gastric cancer tissues. THBS1 enhances the cytotoxic activity of Vγ9Vδ2 T cells against gastric cancer cells via an m6A-dependent activation of the RIG-I-like Receptor (RLR) Signaling Pathways, concomitantly upregulating Interferon-γ (IFN-γ), Interferon-α (IFN-α), perforin and granzyme B. Recent studies have also shown that circMAN1A2 in exosomes derived from gastric cancer cells is highly expressed. Upon binding to the proline- and glutamine-rich splicing factor SFPQ in both gastric cancer cells and T cells, circMAN1A2 promotes G1/S-phase progression in gastric cancer cells while inhibiting T-cell receptor (TCR) signaling and the secretion of TNF-α and IFN-γ, thereby attenuating antitumor immune responses and driving gastric cancer progression (69).

#### 2.2.3 Spatial distribution of metabolites

Wang et al. (70) ‘s study employed spatial metabolomics based on mass spectrometry imaging (MSI) to analyze gastric cancer patients, established metabolic classifications for tumor- and matrix-specific tissue regions, and identified three tumor-specific subtypes with distinct tissue metabolite patterns as well as three matrix-specific subtypes, thereby revealing the metabolic differences among these subtypes and their underlying molecular characteristics.

A study (71) employing spatial metabolomics, spatial lipidome, and spatial transcriptome analyses showed that glutamine levels in peritumoral lymphoid tissue (PLT) of gastric cancer were significantly downregulated, whereas glutamate levels were markedly higher than in distal lymphoid tissue (DLT). In addition, the GLS gene, which regulates the conversion of glutamine to glutamate, and the glutamine transporter SLC1A5 were highly expressed in PLT. These observations indicate that glutamine in PLT is over-absorbed and consumed, underscoring its important role in gastric cancer TIME. Furthermore, excessive oxidation within tumor tissue led to significant downregulation of histamine, and unsaturated FA displayed spatially heterogeneous distribution. Notably, within the elongated, narrow “tumor border area” enriched in immune cells, there was pronounced metabolic reprogramming: immune cells exhibited upregulated glutamine metabolism and polyunsaturated fatty acid expression, suggesting that tumor immune escape may be closely linked to these metabolic alterations.

These research methods and findings provide cutting-edge new ideas for exploring the precise spatial localization of metabolites, lipids and gene expression characteristics in gastric cancer TIME.

## 3 Traditional treatments

Surgical resection remains the cornerstone of curative therapy for gastric cancer and effectively controls localized disease, but its benefits are limited in advanced or metastatic cases. In early-stage gastric cancer, endoscopic mucosal resection (EMR) and endoscopic submucosal dissection (ESD) serve as the primary treatment modalities (72). Chemotherapy can reduce tumor size to facilitate surgical resection and eradicate or suppress micrometastases, thereby improving patient survival. However, it carries significant adverse effects. For potentially resectable patients with clinical T2N0 or greater disease, neoadjuvant or perioperative therapy is preferred over upfront surgery followed by adjuvant treatment. Indeed, perioperative chemotherapy has become the standard of care for resectable, localized gastric cancer (6, 13). The phase 2/3 FLOT4-AIO trial compared perioperative FLOT (fluorouracil plus leucovorin, oxaliplatin, and docetaxel) to ECF (or ECX where X refers to capecitabine) in patients with resectable gastroesophageal adenocarcinoma, and established FLOT as the new standard of care (73).

### 3.1 Adjuvant chemotherapy

In patients with gastric cancer who undergo upfront surgery and have pathological T3 or T4 lesions, or node positive disease, adjuvant therapy is recommended (13). The CLASSIC trial established the benefit of adjuvant capecitabine and oxaliplatin in patients who undergo curative-intent gastrectomy with D2 (extended) lymph node dissection (74).

### 3.2 Adjuvant chemoradiotherapy

Chemoradiotherapy was once the preferred approach for resectable gastric cancer, yet the role of radiotherapy in the adjuvant setting remains contentious (6). Results from trials such as CRITICS (75) and ARTIST-2 (76) demonstrated no benefit from postoperative radiotherapy, even in high-risk patients. Consequently, routine use of adjuvant radiotherapy is no longer recommended (except in cases of D0 or D1 lymph node dissection or R1 resection).

### 3.3 Preoperative chemoradiotherapy

Preoperative chemoradiation is a category 2B (based upon lower-level evidence) treatment option for patients undergoing a preoperative therapy or total neoadjuvant treatment approach (13). The NCT01924819 (77)clinical trial demonstrated that, in patients with resectable gastric adenocarcinoma or gastroesophageal junction adenocarcinoma, the addition of preoperative radiotherapy to chemotherapy did not improve overall survival compared with perioperative chemotherapy alone.

### 3.4 Hyperthermic intraperitoneal chemotherapy

Data from the Phase III DRAGON-01 (78) trial demonstrated that, in patients with gastric cancer and peritoneal metastases, the intraperitoneal plus intravenous paclitaxel combined with S-1 (NIPS) regimen significantly prolonged OS compared to the intravenous paclitaxel plus S-1 (PS) regimen alone, with manageable toxicity. This study was the first to confirm the significant efficacy of intraperitoneal normothermia combined with systemic therapy (NIPS) in this patient population and has established NIPS as a consensus treatment approach for gastric cancer with peritoneal metastases in Asia.

With anti-HER2 and anti-vascular endothelial growth factor (VEGF) therapies established as standard treatments for gastric cancer, programmed death 1 (PD-1) inhibitors have also been approved in multiple countries for the first-line treatment of unresectable or metastatic disease. Research on targeted therapy and immunotherapy directed at the TIME is progressing steadily, marking the formal entry of TIME-targeted strategies into the therapeutic landscape of gastric cancer (3).

## 4 Intervention strategies targeting the TIME

With the deepening understanding of the immunosuppressive microenvironment in gastric cancer, the field of gastric cancer treatment is undergoing accelerated innovation. Immune checkpoint inhibitors combined with chemotherapy have significantly prolonged patient survival, while targeted therapies, dual immune blockade, and triple-combination regimens are showing breakthrough potential. Emerging approaches such as CAR-T cell therapy, oncolytic viruses, and metabolic interventions are expected to overcome drug resistance, whereas cancer vaccines and cell therapies are advancing individualized and precision treatment. Under the synergy of multiple strategies, gastric cancer therapy is steadily moving toward a new paradigm characterized by high efficacy and low toxicity (**Table 1**).

**Table 1:** Clinical trials investigating the TIME in gastric cancer.

Drug combination	Mechanism of additional agents	Chemotherapy	ICB	Phase	Population	Clinical trial
Nivolumab	NO	Yes	PD-1	3	NonHER2-positive advanced GC/GEJC/EAC	NCT02872116
Sintilimab	NO	Yes	PD-1	3	Unresectable locally advanced or metastatic gastric and gastroesophageal junction adenocarcinoma	NCT03745170
Toripalimab	NO	Yes	PD-1	2	Locally advanced gastric or gastro-esophageal junction cancer	NCT04250948
Zolbetuximab	Zolbetuximab: CLDN18.2 inhibitor	Yes	NO	3	CLDN18.2-positive, HER2-negative gastric or gastroesophageal junction adenocarcinoma	NCT03653507 NCT05014060
IBI110 Sintilimab	NO	Yes	PD-1 、LAG-3	1	Advanced HER2-negative gastric cancer or gastroesophageal junction cancer	NCT04085185
Cadonilimab	NO	Yes	PD-1 、CTLA-4	3	Untreated, unresectable, locally advanced or metastatic G/GEJ adenocarcinoma	NCT05008783
Ienvatinib Pembrolizumab	Lenvatinib: Multi-kinase inhibitor	Yes	PD-1	3	Untreated, HER2-negative, locally advanced unresectable or metastatic gastroesophageal adenocarcinoma	NCT04662710
Stiripentol immune-targeted	Stiripentol: LDHA inhibitor	Yes	Not available	1	Peritoneal metastatic carcinoma refractory	ChiCTR2400083649
Regorafenib Nivolumab	Regorafenib: Multi-kinase inhibitor	NO	PD-1	3	Refractory advanced gastric and esophagogastric junction cancer (AGOC)	NCT04879368
CDK-004	CDK-004: STAT6 inhibitor	NO	NO	1	Liver Metastases From Either Primary Gastric Cancer	NCT05375604
Domvanalimab Zimberelimab	Domvanalimab: TIGIT inhibitor	Yes	PD-1	2	Previously untreated G/GEJ/E adenocarcinoma	NCT05329766
GEN-001 Avelumab	GEN-001: Targeting the microbiome	NO	PD-L1	2	PDL1-positive GC	NCT05419362
Neo-MoDC Nivolumab	Neo-MoDC: Personalized neoantigen-loaded monocyte-derived dendritic cell vaccine	NO	PD-1	1	Metastatic gastrointestinal cancer	NCT03185429
VG161 Nivolumab	VG161: Recombinant Human IL12/15-PDL1B Oncolytic HSV-1 Injection (Vero Cell)	NO	PD-1	2	Advanced metastatic gastric or gastroesophageal junction adenocarcinoma who have previously received two or more systemic treatment regimens (including anti-PD-1 monoclonal antibodies)	NCT06008925
ASKB589 PD-1 Inhibitor	ASKB589: CLDN18.2 inhibitor	Yes	PD-1	3	Advanced G/GEJ cancer with CLDN18.2 positive	NCTo5632939
AZD5863	AZD5863: CLDN18.2 and CD3 inhibitor	NO	NO	1/2	Advanced or Metastatic Solid Tumors	NCT06005493
ASP2138 Pembrolizumab	ASP2138: CLDN18.2 and CD3 inhibitor	NO	PD-1	1	Metastatic or Locally Advanced Unresectable Gastric or Gastroesophageal Junction (GEJ) Adenocarcinoma	NCT05365581
Avelumab Ramucirumab	Ramucirumab: VEGFR-2 inhibitor	Yes	PD-L1	2	Adenocarcinoma of the gastro-esophageal junction or the stomach who have documented progression after being treated with a 1st line chemotherapy which contained at least a platinum and 5-FU (5-Flourouracil)	NCT03966118
CT041/satri-cel PD-1 Inhibitor	CT041 autologous CAR-T targeting CLDN18.2	NO	PD-1	1	Untreated, CLDN18.2-positive solid tumors	NCT03874897
KACM001	KACM001: Autologous lymphocytes	Yes	NO	2	Stomach/gastroesophageal junction adenocarcinoma	Not available
CYNK-101 Trastuzumab Pembrolizumab	CYNK-101: NK cell product, a variant of CD16, Fc gamma receptor III (FcγRIII)	Yes	PD-1	2	Locally advanced unresectable or metastatic HER2-Positive Gastric or Gastroesophageal junction (G/GEJ) adenocarcinoma	NCT05207722
Durvalumab	NO	Yes	PD-L1		gastric and gastroesophageal junction cancer	NCT04592913

### 4.1 Optimization of traditional combination therapy

Traditional combination therapies typically involve surgery, chemotherapy, and radiotherapy. With the advent of targeted therapy and immunotherapy, new combination treatment strategies have emerged, expanding the options beyond conventional approaches.

Common immune checkpoints in gastric cancer include PD-1/PD-L1 and CTLA-4, whereas emerging targets encompass LAG-3, TIM-3 and CLDN18.2, among others. Immune checkpoint inhibitors (ICIs) targeting PD-1 and CTLA-4 activate CD8^+^ T cells and augment their antitumor immune response. Moreover, chemotherapeutic agents can induce immunogenic cell death of tumor cells, and when combined with ICIs they act synergistically to enhance antitumor immunity (79). This combination strategy not only optimizes traditional combination therapy but also reshapes the gastric cancer TIME more effectively.

#### 4.1.1 Single-agent immunotherapy combined with chemotherapy

Nivolumab (80, 81) is the first PD-1 inhibitor administered in combination with chemotherapy to demonstrate superior overall survival (OS) and progression-free survival (PFS), as well as an acceptable safety profile, compared with chemotherapy alone. The Phase III ORIENT-16 study (82) represents a new milestone in immunotherapy. Sintilimab combined with XELOX (oxaliplatin and capecitabine) significantly extends OS in patients with advanced gastric cancer: in the full study population, median OS increased by 2.9 months—to 15.2 months—and the PD-L1-positive population also achieved a 5.5-month improvement. This accomplishment has been incorporated into the CSCO Guidelines for the Diagnosis and Treatment of Gastric Cancer. In the NCT04250948 trial, perioperative administration of toripalimab combined with chemotherapy significantly increased the proportion of patients achieving tumor regression grade 0 or 1 (TRG0/1) from 20.0% to 44.4% and raised the pathological complete remission rate from 7.4% to 22.2%, without increasing surgery- or treatment-related adverse events, thereby offering a safe and effective new perioperative immunotherapy protocol for patients with locally advanced gastric cancer (83).

In the recent NCT04592913 study, perioperative Durvalumab combined with the FLOT regimen significantly improved event-free survival in patients with gastric and gastroesophageal junction cancer. The latest 5-year data from the CheckMate-649 Chinese subgroup have set a new survival milestone, marking the first time that the goal of “chronicizing” advanced gastric cancer has been proposed, with over 20% of patients projected to achieve long-term survival.

#### 4.1.2 Targeted therapy combined with chemotherapy

CLDN18.2 is a tight-junction molecule predominantly expressed in non-malignant gastric epithelium and becomes exposed on the surface of tumor cells during malignant transformation. Results from the Phase III GLOW and SPOTLIGHT studies both demonstrated that the chemotherapy regimen including zolbeximab conferred clinically meaningful PFS and OS benefits in Chinese patients with advanced gastric cancer (GC) or gastroesophageal junction cancer (GEJC) who were CLDN18.2-positive and HER2-negative, with a favorable safety profile. The latest results show that Zolbeximab has now been approved for clinical use (84–86).

#### 4.1.3 Dual immunotherapy combined with chemotherapy

In addition, dual immune-checkpoint blockade, either alone or in combination with chemotherapy, has led to significant advances in gastric cancer treatment. Studies have shown that Lymphocyte-activation gene 3 (LAG-3) and PD-1 synergistically engage CD8^+^ T cells, promoting T cell depletion. The latest research progress is as follows:

Dual inhibition of PD-1 and LAG-3 may further enhance the antitumor effect (87). The ongoing study of IBI110 (IgG4 κ-type recombinant fully human anti-LAG-3 monoclonal antibody) in combination with sinidimab and XELOX in first-line gastric adenocarcinoma patients has demonstrated a favorable safety profile and promising efficacy results (88).

Wang et al. (89)’s analysis indicated that combination therapy with PD-1 and CTLA-4 inhibitors elicited more robust multi-clonal responses of tumor-specific and depleted CD8^+^ T cells. Moreover, αCTLA-4 promoted the expansion of progenitor-like depleted T cells, whereas αPD-1 tended to induce their differentiation. Recent findings from the COMPASSION-15 study (90) suggest that, compared with chemotherapy alone, the PD-1/CTLA-4 bispecific antibody Cadonilimab combined with chemotherapy significantly improves progression-free and overall survival in patients with previously untreated, HER2-negative, locally advanced or metastatic gastric or gastroesophageal junction (GEJ) cancer, including those with low PD-L1 expression.

Recent studies have shown that simultaneous inhibition of T-cell immunoreceptor with Ig and ITIM domains (TIGIT) and PD-L1 can promote the CD226-driven expansion of tumor-reactive CD8 T cells from tumor-draining lymph nodes (TDLNs) into the peripheral blood, followed by their infiltration into tumor sites. This combination also establishes favorable co-stimulatory conditions that facilitate the differentiation of tumor-reactive CD8 T cells into an effector rather than an exhausted phenotype, thereby enhancing their anti-tumor activity (91).

In the EDGE-GastricArM A1 study, domvanalimab (D) and zimberelimab (Z), in combination with FOLFOX as first-line treatment for advanced gastroesophageal cancer, demonstrated a high objective response rate and median progression-free survival, with improved outcomes in patients with high PD-L1 expression and a favorable tolerability profile. Ongoing investigations are also evaluating various dual immune checkpoint inhibitors or their combinations with chemotherapy, such as lenvatinib plus pembrolizumab with chemotherapy (NCT04662710) (92).

### 4.2 Innovative therapies

Lactic acid, amino acids, exosomes, and microbial metabolism within the tumor immune microenvironment of gastric cancer all contribute to the development of immunosuppression, making them potential targets for therapeutic intervention. Research in this area is steadily advancing, and a variety of innovative treatment strategies are beginning to emerge.

#### 4.2.1 Anti-lactate therapy

Studies have demonstrated that the anticonvulsant Stiripentol effectively inhibits lactic acid production in gastric cancer cells and suppresses the lactylation of NBS1K388, thereby reducing DNA repair efficiency and overcoming tumor resistance to chemotherapy and radiotherapy. Moreover, its combination with cisplatin or ionizing radiation (IR) exhibits strong synergistic effects, positioning it as one of the most promising lactate dehydrogenase A (LDHA) inhibitors in current research (53).

#### 4.2.2 Tyrosine kinase inhibitor therapy

Regorafenib (Rego) is an oral multi-target tyrosine kinase inhibitor (TKI) that exerts its effects by targeting angiogenesis, matrix kinases, and receptor tyrosine kinases. In the INTEGRATE IIA study, Rego significantly improved survival in patients with refractory advanced gastric and esophagogastric junction cancer (AGOC) compared with placebo (93). The subsequent INTEGRATE Iib study further evaluated the efficacy and safety of Rego in combination with nivolumab.

#### 4.2.3 Exosome-based therapy

A 22-year phase I study investigating the macrophage reprogramming agent exoASO-STAT6 (CDK-004) in patients with advanced solid tumors, including gastric cancer, was initiated. CDK-004 is composed of cell-derived exosomes loaded with synthetic lipid-labeled oligonucleotides. It is designed to specifically deliver STAT6 antisense oligonucleotides (ASO) to myeloid cells, thereby promoting the M2-TMAs phenotype and re-polarizing M1-TAMs. In multiple *in vivo* preclinical studies, CDK-004 demonstrated potent single-agent activity, inhibiting over 90% of tumor growth and achieving complete response (CR) rates of 50–80% (94). Although the study was suspended due to funding limitations, it remains of pioneering significance in the field of exosome-based therapy. Additionally, the internally and externally engineered exosome IEEE (also known as I3E), developed by Zhang et al. (95), can accurately and efficiently reprogram TAMs *in situ*, exhibiting strong potential in cancer immunotherapy.

GEN-001 is an innovative oral therapeutic candidate comprising a single strain of Lactococcus lactis. In this study, GEN-001 was administered in combination with Avelumab for the treatment of PD-L1-positive, locally advanced or metastatic gastric or gastroesophageal junction cancer that had progressed following second-line therapy. The combination demonstrated favorable efficacy and safety profiles. This represents the first clinical study to demonstrate the potential of microbiome-based therapy in the treatment of gastric cancer (96).

#### 4.2.4 Cancer vaccine

The cancer vaccine Neo-MoDC is a personalized neoantigen carrier derived from monocyte-derived dendritic cells. A patient with metastatic gastric cancer received Neo-MoDC vaccination and developed a T-cell response targeting a neoantigen. Subsequent combination with immune checkpoint inhibitor therapy elicited a more robust immune response and led to complete tumor regression within 25 months (97). These findings highlight the significant potential of novel combination immunotherapy strategies involving cancer vaccines for the treatment of metastatic gastric cancer.

#### 4.2.5 Oncolytic virus therapy

In addition, oncolytic virus (OV) therapy has demonstrated considerable potential in the treatment of malignant tumors. Zhong et al. (98) have achieved notable advances in the application of OV therapy for refractory hepatocellular carcinoma. Their preclinical and clinical trial results indicate that NDV-GT (genetically engineered oncolytic virus therapy based on Newcastle disease virus) exhibits significant efficacy and favorable safety in refractory cancers, offering novel insights and technical support for the development of OV-based therapies. Currently, combination therapies involving OVs have shown safety and controllability in anal cancer, pancreatic cancer, and other malignancies, and have also demonstrated marked efficacy in non-small cell lung cancer. An ongoing Phase Ib/IIa clinical trial (NCT06008925) is designed to evaluate the efficacy of VG161 in combination with nivolumab injection in patients with metastatic gastric cancer.

### 4.3 Breakthroughs in triple combination therapy

As the latest drug combination model, triple therapy has the following latest developments:

Triple Combination Therapy ASKB589 is a recombinant humanized monoclonal antibody targeting CLDN18.2. It exerts antitumor effects by mediating antibody-dependent cell-mediated cytotoxicity (ADCC) and complement-dependent cytotoxicity (CDC) through high-affinity binding to CLDN18.2-expressing cancer cells. A Phase Ib clinical study evaluating ASKB589 in combination with CAPOX (oxaliplatin and capecitabine) and PD-1 inhibitors as a first-line treatment for patients with locally advanced, recurrent, or metastatic gastric and esophagogastric junction adenocarcinoma represents the first targeted triple immunotherapy regimen. This combination has demonstrated deep tumor regression, durable antitumor activity, and favorable tolerability. The study has now progressed to a pivotal Phase III clinical trial (99, 100). In addition, several other agents targeting CLDN18.2 are under active development, including AZD5863 and ASP2138, both of which are T cell-engaging bispecific antibodies targeting CLDN18.2 and CD3.

In the non-randomized, controlled phase 2 trial (NCT03966118), a triple therapy comprising Ramucirumab, Avelumab, and Paclitaxel was administered as a second-line treatment in patients with esophageal and gastric adenocarcinoma (EGA). The median overall survival was 10.6 months (95% confidence interval: 8.4–12.8 months). Regarding safety, the regimen was well tolerated and holds promise as a novel second-line triple therapy for advanced gastric cancer (101).

### 4.4 Adoptive cell transfer therapy

Adoptive cell transfer therapy (ACT) is an advanced immunotherapeutic approach in which a patient’s immune cells are harvested, expanded, and genetically or functionally engineered *in vitro* before being reinfused to target and eliminate pathogens or malignant cells. Common forms of ACT include T-cell receptor–engineered T-cells (TCR-T) therapy, tumor-infiltrating lymphocyte (TIL) therapy, natural killer (NK) cell therapy, chimeric antigen receptor T-cell (CAR-T) therapy, and cytotoxic T lymphocyte (CTL) therapy. Notably, CAR-T therapy has received regulatory approval and demonstrated remarkable efficacy in the treatment of hematologic malignancies (102).

CT041/Satricabtagene autoleucel (satri-cel) is a CAR-T cell therapy that specifically targets CLDN18.2. An interim analysis of the NCT03874897 trial demonstrated that CT041/satri-cel exhibits a favorable safety profile and promising efficacy in patients with gastric or gastroesophageal junction adenocarcinoma (GC/GEJ) (103). As an emerging modality in biological immunotherapy, Multi-target Cytotoxic T Lymphocytes (MTCA-CTL) immunotherapy not only promotes the expansion of non-MHC-restricted invariant NK-T cells (iNKT), but also selectively directs the proliferation of MHC-restricted, CD8^+^ antigen-specific CTLs, thereby enhancing tumor‐cell cytotoxicity. CYNK-101 is derived from human placental hematopoietic stem cells and genetically engineered to express a high-affinity, non-cleavable variant of CD16 (FCGR3A), thereby potentiating NK-cell–mediated cytotoxicity. The latest progress is as follows:

The most recent data from the CT041-CG4006 study, based on full-population analysis, indicate that CT041/satri-cel therapy maintains a strong safety profile and offers significant therapeutic potential in patients with advanced gastrointestinal malignancies. Moreover, the Phase II confirmatory randomized controlled trial evaluating CT041/satri-cel in third-line and later-line gastric cancer patients is nearing completion (104).

KACM001 (Autologous Lymphocyte Injection) represents an MTCA-CTL therapeutic agent. Clinical data presented at the 2023 ASCO Annual Meeting demonstrated its favorable safety profile and promising efficacy (105). A Phase I/II study has now been initiated to assess the safety, tolerability, and preliminary efficacy of KACM001 in combination with S-1/oxaliplatin or cisplatin in patients with locally advanced unresectable or metastatic gastric cancer.

A Phase I/IIa clinical trial is currently underway to evaluate CYNK-101 in combination with trastuzumab and pembrolizumab in patients with locally advanced unresectable or metastatic HER2-positive gastric or gastroesophageal junction adenocarcinoma (106).

## 5 Outstanding questions and clinical challenges

### 5.1 TIME heterogeneity

#### 5.1.1 Cellular heterogeneity

Despite extensive research elucidating the roles of various cell populations within the gastric cancer TIME and the mechanisms by which they promote tumor progression, our understanding of its full complexity remains limited. What still demands in-depth study is: on one hand, the interactions among distinct heterogeneous cell populations represent a gap in supporting clinical diagnosis and treatment; on the other hand, although single-cell sequencing and spatial transcriptomics can reveal spatiotemporal diversity, can these technologies genuinely transcend current insights into the TIME? Moreover, in the realm of clinical translation, the standard murine models used in research do not directly correspond to human systems, severely constraining safety and efficacy evaluations.

Some studies have shown that CAFs populations foster gastric cancer progression within the microenvironment, while others have identified CAF subtypes that participate in antitumor immune responses. This apparent contradiction lies at the heart of gastric cancer’s high heterogeneity—cellular reprogramming and functional outputs vary by tissue origin and tumor subtype and are dynamically reshaped by microenvironmental signals.

#### 5.1.2 Metabolic heterogeneity

Compared with cellular heterogeneity, metabolic diversity in the gastric cancer TIME is even more intricate. To date, the roles of lactate, lipid and nucleotide metabolic reprogramming, as well as exosome-mediated metabolic signaling, have only been preliminarily explored *in vitro* and in murine models and still demand comprehensive investigation. Metabolism-based molecular classification of gastric cancer likewise warrants deeper study. Clinically, the sheer complexity and structural diversity of metabolites present formidable challenges for precise detection and real-time monitoring. Thus, future efforts must achieve breakthroughs in clinical validation and dynamic monitoring technologies for metabolic biomarkers to underpin gastric cancer diagnosis, prognostic assessment and personalized precision therapy.

### 5.2 Intervention strategies targeting the TIME

#### 5.2.1 Optimization of traditional combination therapy

In immune-chemotherapy combinations, biomarker generalizability remains low, and the interactions with diverse cell populations across different immune microenvironments require further study. In targeted-chemotherapy combinations, issues such as dynamic target loss and compensatory resistance arise. For instance, the Claudin18.2-directed antibody oznecitamab demands strong expression in at least 75% of tumor cells, yet target levels may decline during treatment. Real-time monitoring technologies—such as dynamic ctDNA tracking—are still underdeveloped.

While pursuing novel targets for both targeted and immunotherapies, it is equally critical to optimize existing combination regimens. Rational drug pairings can boost efficacy and reduce toxicity, improving outcomes and minimizing adverse effects to advance precision medicine. However, clinical translation of combination therapies faces significant hurdles: complex protocol design, overlapping toxicities, and imbalanced drug bioavailability must be urgently addressed.

#### 5.2.2 Innovative therapies

Despite the advent of various innovative therapies, they remain in early exploratory stages. Research gaps persist in comparing the efficacy of anti-lactate metabolic interventions across gastric cancer subtypes and their effects on non-tumor tissues. Clinical translation faces multiple hurdles: metabolic regulation therapies struggle with precision, often disrupting systemic energy balance, and precise delivery methods remain a bottleneck. Exosome-based treatments are hindered by challenges in engineering and large-scale production, and high development costs may slow progress. Oral microbiota therapies confront intestinal barrier limitations, individual variability, and difficulties in colonizing engineered strains. Cancer vaccines must overcome the hurdle of inducing T cells to evade Treg suppression, while oncolytic virus therapies need to balance antiviral and antitumor responses and optimize dosing regimens. These represent critical research directions and translational challenges moving forward.

#### 5.2.3 Breakthroughs in triple combination therapy

Triotherapy, a newly emerged treatment modality, demands rigorous safety evaluation. It faces challenges including cumulative toxicity, a lack of scalable biomarkers, and the high cost of personalized regimen design. Clinical translation will require interdisciplinary collaboration and multi-omics integration to devise rational combination strategies and avoid mere additive approaches.

#### 5.2.4 Adoptive cell transfer therapy

Major challenges in adoptive cell therapy for solid tumors include poor immune cell infiltration into tumor tissue and a hostile nutritional and metabolic microenvironment that compromises cell survival. For clinical translation, it is essential to determine whether combination therapies can boost immune cell efficacy or whether leveraging the diverse immune cell populations within the tumor microenvironment can generate synergistic effects to improve treatment outcomes.

## 6 Concluding remarks and future perspectives

Currently, the treatment of gastric cancer remains fraught with challenges, yet targeting the heterogeneous TIME of gastric cancer offers a novel therapeutic paradigm. Unlike conventional modalities, modulation of the cellular constituents and intricate signaling networks within the microenvironment—as well as metabolic pathways of glucose, lipids, and nucleotides—not only reprograms the functionality of immune cells to overcome immune evasion, but also perturbs tumor cell bioenergetics and aberrant spatial organization. Immunotherapy plays a critical role in sculpting the gastric cancer TIME and reversing immunosuppressive states, and it is intimately linked to tumor proliferation, metastasis, and therapeutic resistance. To date, monotherapies targeting individual metabolic axes have demonstrated limited efficacy. Consequently, therapeutic strategies have evolved from traditional combination regimens to emerging dual immune‐checkpoint blockade, triplet therapies, and adoptive cell transfer approaches. These innovative interventions have shown promising antitumor activity in both preclinical models and early-phase clinical trials. A comprehensive dissection of the molecular and metabolic rewiring events, coupled with real-time monitoring of dynamic TIME adaptations, will be essential for optimizing personalized immunotherapeutic regimens and improving patient outcomes—and may also inform treatment strategies across other solid tumor types.
